# In Vitro Effects of Low-Level Laser Therapy on the Viability and Secretory Activity of Resting Macrophages

**DOI:** 10.3390/biomedicines13020403

**Published:** 2025-02-07

**Authors:** Aleksandra Matuła, Amelia Lizak, Ewa Stodolak-Zych, Aneta Bac, Joanna Homa, Beata Stenka, Anna Ścisłowska-Czarnecka

**Affiliations:** 1 Department of Applied Cosmetology, Institute of Applied Sciences, University of Physical Culture, 31-571 Kraków, Poland; aleksandra.matula@awf.krakow.pl (A.M.); amelia.lizak@awf.krakow.pl (A.L.); aneta.bac@awf.krakow.pl (A.B.); 2 Faculty of Materials Science and Ceramics, AGH University of Krakow, 30-059 Kraków, Poland; stodolak@agh.edu.pl; 3 Department of Evolutionary Immunology, Institute of Zoology and Biomedical Research, Jagiellonian University, 31-007 Kraków, Poland; joanna.homa@uj.edu.pl; 4 Department of Applied Cosmetology, University of Physical Education and Sport, 80-854 Gdańsk, Poland; beata.stenka@awf.gda.pl

**Keywords:** macrophages, LLLT, photobiostimulation, viability, secretory activity

## Abstract

**Background/Objectives**: Low-level laser therapy (LLLT) covers a wide range of parameters in terms of laser properties and dosage, which is important for its effects. It is important to select safe, optimal irradiation conditions to obtain the desired therapeutic effect of LLLT on cells. This article is focused on the selection of favourable (biostimulating) exposure conditions for LLLT, which are the beam application method (continuous [C] or pulsed [P] laser beam), radiation power and LLLT dose, on the viability and secretory activity regarding resting macrophages of the RAW 264.7 cell line. **Methods**: RAW 264.7 macrophages were seeded on 24-well tissue culture. ASTAR PhysioGo 400C apparatus with a spot applicator generating electromagnetic radiation in the infrared light range of 808 nm and power of 100 mW and 200 mW was used for laser irradiation of macrophages. Cells were treated with different doses of constant radiation 5 J/cm^2^/well or 10 J/cm^2^/well. **Results**: It was shown that the most beneficial radiation parameters for cells were obtained with a pulsed laser beam of 200 mW power and a dose of 5 J/cm^2^, which caused an increase in macrophage adhesion and viability, as well as an increase in NO secretion by macrophages and their TOS, with a simultaneous decrease in the secretion of TNF-α, MCP-1 and MMP-9 by cells. **Conclusions**: The research results presented above indicate that the effect of LLLT on resting macrophages modulates their biological activity, and the intensity of photobiostimulation depends on the irradiation parameters, including wavelength, power, dose and method of laser beam application.

## 1. Introduction

Low-level laser therapy (LLLT) is becoming an increasingly promising approach in regulating chemical and biochemical agents in the cells of living organisms. It has been shown that irradiation of cells with low-level radiation has a photobiomodulating effect on them, while inducing changes in their viability, application and proliferation [1,2,3,4]. It may also cause changes in the secretory activity of cellular enzymes, measured by, among others, the synthesis of various types of proteins, free radical RONS (reactive oxygen and nitrogen species) or ATP (adenosine triphosphate) [4,5,6,7]. Low-level laser therapy (LLLT) is a fast-growing technology used to treat a multitude of conditions that require biostimulation of the cells. The reparative processes activated by LLLT result in rejuvenating effects, such as wrinkle reduction and skin texture improvement. In other studies, the effectiveness of LLLT was indicated in the treatment of scars or keloids, psoriasis and acne. LLLT covers a wide range of parameters in terms of laser properties and dosage, which is important for its effects. To obtain the desired therapeutic result of LLLT on cells, it is important to select optimal irradiation conditions: radiation wavelength, surface power density, energy supplied and the number of irradiations [1,4,6,7,8,9,10,11,12]. These factors determine what changes will be induced in cells under the influence of LLLT.

Recently, a significant amount of scientific research has been focused on the use of LLLT for photobiostimulation of skin cells, especially fibroblasts, which play a significant role in the regeneration and reconstruction of skin tissues. It has been demonstrated that LLLT parameters that are too low may result in no cellular response or one that is too weak, while using irradiation parameters that are too strong may have a cytotoxic effect on cells or may not initiate a cellular response at the expected level. An example of such behaviour has been described, among others, by (Crisan, 2013), who compared the effects of 830 nm and 980 nm radiation wavelengths on fibroblasts, showing significantly stimulated cell proliferation after one-, two- and also three-fold irradiations [13]. Ma et al. (2018) also described the increased proliferation of human fibroblasts and collagen synthesis under irradiation with a wavelength of 830 nm, while irradiation with a wavelength of 635 nm did not increase fibroblast proliferation and collagen synthesis [14]. Mignon et al. (2018) showed that shorter wavelengths of radiation (530 nm) inhibit the metabolic activity of fibroblasts, while longer wavelengths (550–850 nm) do not produce such an effect [15]. It has been noted that the radiation dose is also a factor that modulates cellular response. Hawkins and Abrahams (2006) treated human skin fibroblasts with radiation doses of 2.5 J/cm^2^, 5 J/cm^2^ and 16 J/cm^2^ for two consecutive days. The results indicated that a dose of 2.5 J/cm^2^ for daily irradiation and 5 J/cm^2^ for single irradiation increased cell viability, proliferation and migration. However, exposure at a dose of 16 J/cm^2^ inhibited cell proliferation, which had a negative effect on viability and migration [16]. In another study, similar findings were achieved, demonstrating that laser irradiation at doses of 3 J/cm^2^ and 4 J/cm^2^ for six consecutive days resulted in increased fibroblast proliferation compared to the control group, while a dose of 5 J/cm^2^ did not stimulate cell proliferation [17]. The power of the laser radiation is also a factor influencing the effectiveness of the cellular response. Azevado et al. (2006) exhibited that the proliferation of fibroblasts increased after two- and six-fold irradiations of cells at a power of 10 mW and 29 mW [18]. Illescas-Montes et al. (2017) observed an increase in the number of cells at an irradiation power of 0.2 W and 0.5 W, while the use of an irradiation power at the level of 1 W did not affect the number of cells [19]. In turn, Chen et al. (2009) presented a decrease in the viability of human fibroblasts after irradiating cells with a laser at powers between 1.0 and 3.0 W [20].

However, there is still relatively little information available regarding the effect of LLLT on the response of immune cells, including macrophages, the activation of which plays a key role in the initiation and course of inflammation, including the healing process [21,22,23]. Macrophages express many receptors and signalling molecules, and their activation depends on local conditions in the tissue. Macrophages often have opposing functions; hence, they are divided into classically polarised M1 and alternatively polarised M2 [23,24]. M1 macrophages mainly occur at the early stages of inflammation. The molecules stimulating their polarisation towards M1 macrophages are LPS (lipopolysaccharide), IFN- and TNF-α [24]. In M1 macrophages, pro-inflammatory signalling pathways are activated, and the factors secreted by them are free radicals—RONS (reactive oxygen and nitrogen species)—and pro-inflammatory cytokines (TNF-α, IL-1 and IL-12p70). In turn, in the processes ending the inflammatory reaction and promoting tissue remodelling, M2 macrophages are mainly involved [24,25,26]. Cells polarise towards M2 macrophages as a result of the action of cytokines such as IL-4 and IL-13, and the factors secreted by them are IL10, TGF-β and metalloproteinases (MMPs), which are associated with the remodelling of the extracellular matrix (ECM) [25].

Prolonged inflammation leads to impaired healing because macrophage dysfunction can result in excessive production of pro-inflammatory cytokines and free radicals, as well as the inability to adequately remove neutrophils. It has been indicated in clinical studies that photobiostimulation reduces tissue inflammation and accelerates wound closure, but precise laser settings to optimise macrophage behaviour have not been established. The development of inflammation during skin healing is largely dependent on the function of resident macrophages because the healing process begins before a full inflammatory response has developed.

Available literature data concerning the effects of LLLT on macrophages mainly concern the response of M1 inflammatory-stimulated (LPS stimulated) macrophages. Due to the fact that the healing process begins before a full inflammatory response develops, understanding how resting cells respond to LLLT will help assess whether a given therapy is safe for cells and able to support tissue regeneration/repair. This article is focused on the selection of favourable (biostimulating) exposure conditions for LLLT, which are the beam application method (continuous [C] or pulsed [P] laser beam), radiation power and LLLT dose, on the viability and secretory activity regarding resting macrophages of the RAW 264.7 cell line.

## 2. Materials and Methods

### 2.1. Macrophage Culture Conditions

The RAW 264.7 macrophage cell line (ATCC, Manassas, VA, USA) was used in this study. The cells were cultured in an RPMI 1640 culture medium (Lonza, Morristown, NJ, USA) with the addition of 10% FBS calf serum (Gibco, Norristown, PA, USA) and a 1% solution of antibiotics—penicillin and streptomycin (Sigma-Aldrich, St. Loius, MO, USA)—in an atmosphere of 5% CO_2_ and at a temperature of 37 °C. Then, 1 mL of the cell suspension at a concentration of 1.5 × 10^4^ cells/mL was placed in the wells of a 24-well culture plate (Nest SB, New York, NY, USA) at the bottom of which round culture slides had previously been placed.

### 2.2. Low-Level Laser Therapy

The PhysioGo 400C device (ASTAR, Bielsko-Biała, Poland) was used to irradiate the macrophages. This is a low-level laser (LLL) that generates electromagnetic radiation in the infrared light range at a wavelength of 808 nm, power of 100 mW or 200 mW and radiation doses of 5 J/cm^2^/well with cells or 10 J/cm^2^/well with cells. The laser beam was continuous (C) or pulsed (P) (at a frequency of 100 Hz with a 50% fill factor). Irradiation was performed using a non-contact method, at a distance of 1 cm from the cells and at a right angle to the irradiated surface.

### 2.3. Study Stages

**Figure 1:**
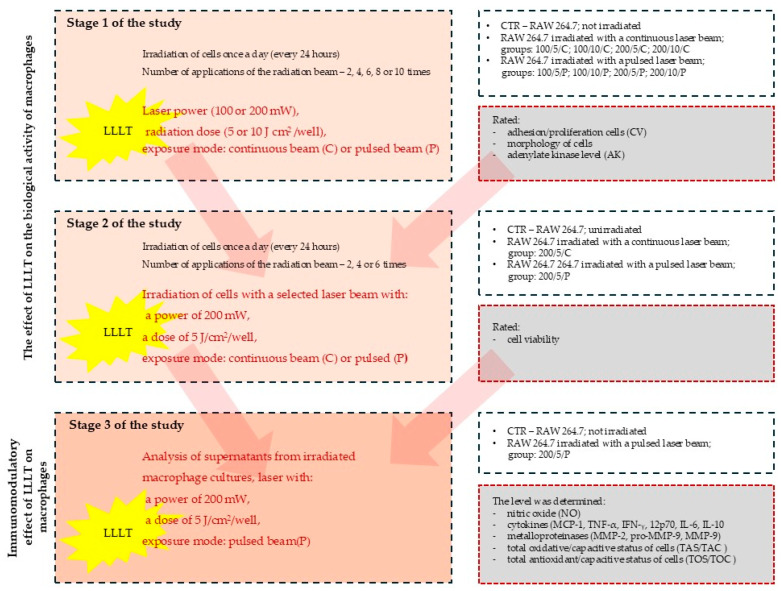
Research design regarding impact of LLLT on macrophage of RAW 264.7 cell line.

The study was carried out in three stages (Figure 1). In stage 1 of the study, laser radiation was applied once a day (at the same time of day), two, four, six, eight or ten times. On the following days of the experiment, on day 3 (two laser beam applications), on day 5 (four laser beam applications), on day 7 (six laser beam applications), on day 9 (eight laser beam applications) and on day 11 (ten laser beam applications), the macrophage culturing was ended and the cells and cell culture supernatants were assessed for adhesion/proliferation, morphology and the level of adenylate kinase (AK) released from dead cells (a test assessing the cytotoxic effect of LLLT on cells).

Stage 1 of the study was carried out for the following groups:

  (i) Unirradiated cells (CTR group–control group);
 (ii) Cells irradiated with a continuous laser beam (C) at powers of 100 mW or 200 mW and doses of 5 J/cm^2^ or 10 J/cm^2^ (100/5/C, 100/10/C, 200/5/C and 200/10/C groups);
(iii) Cells irradiated with a pulsed laser beam (P) at powers of 100 mW or 200 mW and doses of 5 J/cm^2^ or 10 J/cm^2^ (100/5/P, 100/10/P, 200/5/P and 200/10/P groups).

The experimental data made it possible to answer the question of how repeated (eleven times) exposure to LLLT with precisely defined parameters (power, intensity and exposure frequency) affects resting macrophages.

In stage 2 of the study, radiation doses of a specific power that had a biostimulating effect on the cells were used. The cells were irradiated with a continuous (C) or pulsed (P) laser beam at a power of 200 mW and a dose of 5 J/cm^2^. Laser radiation was applied once a day (at the same time of day), two, four or six times. On the following days of the experiment, on day 3 (two laser beam applications), on day 5 (4 laser beam applications) and on day 7 (six laser beam applications), the macrophage culture was ended and the cells and cell culture supernatants were assessed for the functional state of macrophages by determining their viability.

Stage 2 of the study was carried out for the following groups:

  (i) Unirradiated cells (CTR group);
 (ii) Cells irradiated with a continuous (C) laser beam at a power of 200 mW and dose of 5 J/cm^2^ (200/5/C group);
(iii) Cells irradiated with a pulsed (P) laser beam at a power of 200 mW and dose of 5 J/cm^2^ (200/5/P group).

During stage 3 of the study, the effect of LLLT on the secretory activity of macrophages was assessed on the supernatants collected from the cell cultures irradiated with a pulsed (P) laser beam at a power of 200 mW and dose of 5 J/cm^2^ (200/5/P group). In this group, the metabolic activity of the macrophages was examined by determining the levels of nitric oxide (NO), cytokines (MCP-1), tumour necrosis factor TNF-α, interferon gamma IFN-γ, interleukin 12p70, interleukin 6, interleukin 10, metalloproteinases (MMP-2 and MMP-9), total oxidative/capacitive status (TOS/TOC) and total antioxidant/capacitive status (TAS/TAC) for the cells.

### 2.4. Cell Adhesion/Proliferation: Crystal Violet Uptake Assay

The mass of adhered cells was measured by means of the crystal violet test (CV), according to the modified method previously described [27]. Briefly, cells adhered to the bottom of the culture plate were fixed for five minutes with 1 mL 2% paraformaldehyde (PFA) (Sigma, St. Loius, MO, USA), stained for five minutes with a 0.5% CV solution (Sigma, St. Loius, MO, USA), and then rinsed with tap water and air. Then, the dye absorbed by the macrophages was extracted by adding 0.5 mL of 100% methanol (Linegal Chemicals, Blizne Łaszczyńskiego, Poland) to each well. The optical density (OD) of the fluid was read using a FLUOstar Omega reader (BMG Labtech, Ortenberg, Germany) at a wavelength of 570 nm.

### 2.5. Cell Morphology

Some of the tested samples (two from each series) were intended for microscopic observation. Adherent cells were stained for 15 s with a 0.5% solution of CV in water (Sigma, St. Loius, MO, USA) and then rinsed with water. Cells were observed using a light-inverted microscope (Motic AE-2000T, Wetzlar, Germany) at 40× total magnification (objective lens magnification (10×) × eyepiece magnification (4×). Images of cells adhering to the substrate were taken with a Moticam-BTU8 microscope camera (MoticEurope, Barcelona, Spain).

### 2.6. Level of Released AK (ToxiLight Assay)

The level of AK was determined in the tested cell culture supernatant samples according to the instructions provided by the kit manufacturer (ToxiLight assay, Lonza, Switzerland) and previously described [28]. Briefly, first, 20 µL of the supernatant was collected from the cell culture and transferred to a white 96-well plate (Nest SB). Then, 100 µL of AK detection reagent solution was added to each well. After five minutes of incubation at room temperature, luminescence was read using the FLUOstar Omega reader (BMG Labtech). The culture medium served as a negative control, while cells treated with an active AK detection reagent were used as a positive control.

### 2.7. Cell Viability (ViaLight Assay)

Cell viability was determined in the tested cell culture supernatant samples according to the instructions provided by the kit manufacturer (ViaLight assay, Lonza, Switzerland) and previously described [29]. Briefly, first, 200 µL of a cell lysis reagent was added to the wells containing cells and 600 µL of the supernatant. After 10 min of incubation, 200 µL of the supernatant–lyser mixture was transferred to a white 96-well plate (Nest SB) and 200 µL of the AMR PLUS reagent was added. After two minutes, the amount of radiation emitted was determined using the FLUOstar Omega reader (BMG Labtech).

### 2.8. Level of Secreted NO (Griess Test)

Nitrite/nitrate production, an indicator of nitric oxide synthesis, was measured in the cell culture supernatants, as previously described [30]. Briefly, first, 100 μL of the cell culture supernatant was transferred to each well of a 96-well plate (Nest SB). Then, 100 μL of the reagents (Sigma-Aldrich, Hamburg, Germany), Griess A (1% sulfalamide in 5% phosphate acid) and B (0.1% naphthylenediamine in H_2_O), mixed at a 1:1 ratio, was added. After five minutes, the optical density of the liquid was read at a wavelength of 540 nm using the FLUOstar Omega reader (BMG Labtech).

### 2.9. Level of Secreted Cytokines

Cytokine levels in the cell culture supernatants were measured via flow cytometry using the Flex Set (CBA, BD Biosciences, Franklin Lakes, NJ, USA), as showed and described earlier [29,31]. The entire assay procedure as well as all measurements and analyses were performed following the instructions attached to the cytokine assay kit using a Beckman Coulter flow cytometer (Life Science, Boston, MA, USA). The Mouse Inflammation Kit (BD Biosciences, USA) was used, which allows simultaneous determination of the level of six cytokines: interleukin (MCP-1), tumour necrosis factor (TNF-α), interferon gamma (IFN-γ), interleukin 12p70 (IL-12p70), interleukin 6 (IL-6) and interleukin 10 (IL-10). First, 50 µL of a solution, containing a mixture of beads coated with antibodies directed against each of the six analysed cytokines, was added to each Eppendorf tube (Nest SB) containing the standard, a sample or the control. In the next step of the procedure, standards or samples were added to the Eppendorf tubes and an assay diluent was added to the negative control. Then, 50 µL of phycoerythrin (PE)-conjugated antibodies, directed against each of the six cytokines, were added to all Eppendorf tubes and the samples were then incubated in the dark for two hours at room temperature (RT). After this time, 1 mL of washing buffer was added to each sample and they were centrifuged (200 g, five min, RT). The supernatant from the pellets was collected using a pipette and 300 µL of washing buffer was added to each sample. Then, the samples were transferred to new Eppendorf tubes (Nest SB), in which measurements were performed by a cytometer. The flow cytometer was calibrated using BD CaliBRITE™ beads, which are microspheres coated with fluorescein isothiocyanate (FITC) (FL-1), PE (FL-2) and a peridinin–chlorophyll protein complex (PerCp) (FL-3). Consequently, the device was calibrated using CytExpert Software Version 4.2 for the CytoFLEX Platform with the Mouse Inflammation Kit using positive controls for FITC and PE detectors. The data were analysed and cytokine concentrations were determined in Microsoft Excel using standard curves based on successive dilutions of the standard. The maximum concentration of the cytokine standard was 5000 pg/mL. Subsequent serial dilutions were made in the assay diluent at 1:2, 1:4 and 1:8, respectively, ending with a dilution of 1:256, which corresponds to a cytokine concentration of 20 pg/mL.

### 2.10. Level of Secreted Metalloproteinases

The level of metalloproteinases secreted by the cells (in the form of an inactive enzyme precursor and active enzyme) was measured via the gelatin zymography method, which is a modified electrophoretic method that allows the measurement of the proteolytic activity of enzymes, the substrate of which can be incorporated in a polyacrylamide gel with the addition of sodium dodecyl sulphate (SDS) [32]. This method allows detection of the pro-metalloproteinases 9 (pro-MMP-9) and 2 (pro-MMP-2) levels, as well as metalloproteinases 9 (MMP-9) and 2 (MMP-2). First, solutions of separating (10%) and thickening gels (4%) were prepared. Polymerisation catalysts (TEMED, APS) were added immediately before pouring the gels. The separating gel was poured first and some space was left for the thickening gel, in which, after pouring, combs for the wells were placed. Post-solidification (after approx. one h), the gels were placed in a refrigerator for 12 h at 4 °C. To quantitatively analyse the protein levels in the samples, the BCA assay (bicinchoninic acid assay) was performed. Based on the obtained results, protein levels were normalised in the tested samples. Samples with the same protein content were used for further analyses. First, 10 μL of the protein standard with a wide range of proteins (coloured with SDS-PAGE; Bio-Rad, Hercules, CA, USA) was added to the first well and 15 μL of the tested samples was added to the remaining wells. Electrophoresis was performed for 45 min. After electrophoresis was completed, the gels were collected and then washed (Elpan shaker, Lubawa, Poland) twice for 15 min in a 2.5% Triton X-100 solution (Bio-Rad, USA) to remove the SDS (sodium dodecyl sulphate). After this, the gels were placed in an incubation buffer with the following composition, 5 mM CaCl_2_, 50 mM Tris-HCl (pH = 8) and 0.02% NaN_3_ 1 μM ZnSO_4_ (all from Sigma-Aldrich, Hamburg, Germany), and incubated for 24 h in a water bath at 37 °C. Then, the gels were stained in 0.5% brilliant blue solution (Sigma-Aldrich, Hamburg, Germany) and destained in an equilibration buffer until light bands were visible. Syngen’s Snaap Gene and GeneTools software were used to analyse the obtained scans. The average areas of the peaks corresponding to metalloproteinases in the samples were calculated and expressed as raw mean values. The presence of gelatinases was determined based on the molecular masses of the decolourised bands read from the protein standard.

### 2.11. Measurement of Total Oxidative/Capacitive Status (PerOx [TOS/TOC] Assay)

The TOS of the cells was determined by measuring lipid peroxide levels according to the instructions of the PerOx (TOS/TOC) kit (Immunodiagnostik AG, Bensheim, Germany). Peroxide levels in the tested cell culture supernatant samples were established based on the reaction of horseradish peroxidase with tetramethylbenzidine dichloride (TMB) in the presence of hydrogen peroxide. The reaction with the enzyme produces a soluble blue product. The addition of 2 M H_2_SO_4_ stops the reaction, leading to the solution changing colour from blue to yellow. In accordance with the kit instructions, 10 μL of calibrator (CAL), controls 1 and 2 (CTRL1 and CTRL2) (reagents included in the kit) or the tested sample was added to the wells of a 96-well plate (included in the kit). Then, 100 μL of the buffer (Reabuf A) included in the kit was added to each well of the plate and the first OD reading was taken at a wavelength of 450 nm, using the FLUOstar Omega reader (BMG Labtech). Furthermore, 100 μL of the buffer mixture (RBF) (reagent included in the kit) was added to all wells and, in the next step, the plate was incubated for 15 min at 37 °C. After this, 50 μL of the reaction-stopping reagent Stop Solution was added to each well and the second OD reading was taken, also at a wavelength of 450 nm, using the FLUOstar Omega reader (BMG Labtech). Based on the measured optical OD values, the total peroxide level (μmol/L) was determined in the tested cell culture supernatant samples, according to the instructions provided by the kit manufacturer (Immunodiagnostik AG, Bensheim, Germany).

### 2.12. Measurement of Total Antioxidant/Capacitive Status (ImAnOx [TAS/TAC] Assay)

The TAS of the cells was determined by reacting antioxidants with a predetermined (known) amount of exogenous hydrogen peroxide (H_2_O_2_), according to the protocol of the ImAnOx (TAS/TAC) kit (Immunodiagnostik AG, Bensheim, Germany). In this assay, antioxidants react with peroxide and the amount of unreacted H_2_O_2_ is measured spectrophotometrically. The difference between the added and measured amount of H_2_O_2_ (relative to the calibrator included in the kit) is proportional to the antioxidant activity. First, 10 μL of calibrator (CAL), controls 1 and 2 (CTRL1, CTRL2) (reagents present in the kit) or the test sample was added to the wells of the 96-well plates included in the ImAnOx kit. Then, 100 μL of Reagent 1 was added to all wells of the plates and the plates were incubated for 10 min at 37 °C. After the designated incubation time, 100 μL of Reagent 2a was added to all wells of one plate (with enzyme), and Reagent 2b was added to the other plate (without enzyme). Then, both plates were incubated for five minutes at RT, and, following this step, 50 μL of Stop Solution reagent was added to all wells. The OD reading was performed using the FLUOstar Omega reader (BMG Labtech) at a wavelength of 450 nm. Based on the measured OD values, the TAS in the cell culture supernatants was determined, expressed in μmol/L, in accordance with the instructions provided by the kit manufacturer (Immunodiagnostik AG, Bensheim, Germany).

### 2.13. Statistical Analysis

Data are presented as mean values and standard error. Differences between the control group and the experimental groups were established using Student’s *t*-test if the assumptions for parametric tests were met. If the assumptions were not met, the non-parametric Mann–Whitney U test was applied. Differences between the control group and the experimental groups were compared using one-way analysis of variance (ANOVA). Then, Tukey’s test was implemented for post hoc evaluation. A significance level of *p* ≤ 0.05 was adopted in the analyses.

## 3. Results

### 3.1. Adhesion/Proliferation and Morphology of Macrophages Irradiated with LLLT

**Figure 2:**
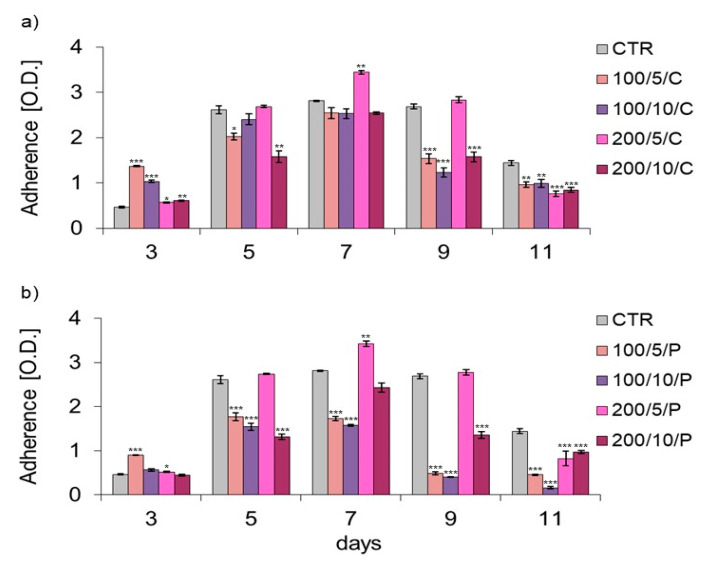
Effect of continuous (C) (**a**) and pulsed (P) laser beam (**b**) on macrophage adhesion of the RAW 264.7 cell line. Cells were cultured for a specified number of days and irradiated with a laser at powers of 100 or 200 mW and radiation doses of 5 or 10 J/cm^2^/cell well. On subsequent days of the experiment (3, 5, 7, 9 and 11), cells were stained with crystal violet. O.D.—optical density was measured at 570 nm. Mean values ± SEM. *, **, ***—differences between cells irradiated with a laser of different parameters and cells not irradiated (CTR) (* *p* < 0.05, ** *p* < 0.01, *** *p* < 0.001).

On the following days of the experiment (days 3–9), unirradiated macrophages (CTR group) proliferated, which resulted in an increased number of adherent cells on days 5, 7 and 9, compared to day 3 of macrophage culturing. On day 11, the number of adherent cells decreased but was still higher than on day 3 of macrophage culturing (Figure 2).

**Figure 3:**
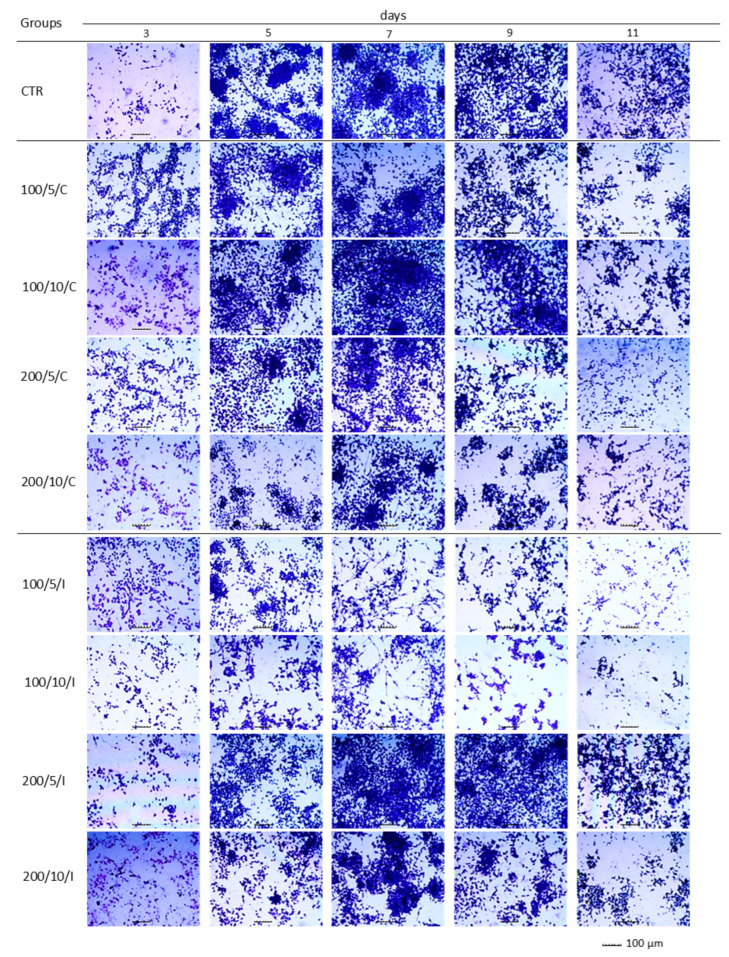
The effect of continuous (C) and pulsed (P) laser beam irradiation on the morphology of the RAW 264.7 cell line macrophages. Cells were cultured for a specified number of days and irradiated with a laser with power of 100 or 200 mW and radiation doses of 5 or 10 J/cm^2^/well with cells. On the following days of the experiment (3, 5, 7, 9 and 11), the cells were stained with crystal violet. Analysis was conducted under a light-inverted microscope at a total magnification of 40× (objective lens magnification 10×) × eyepiece magnification (4×), scale bar 100 μm.

Analysis of the effect of a continuous laser beam on the adhesion/proliferation and morphology of macrophages allowed the conclusion that the two- and six-fold irradiation of cells (days 3 and 7) with a laser at a power of 200 mW and a dose of 5 J/cm^2^ (200/5/C group) resulted in increased adhesion (Figure 2a) and a higher density of macrophages visible in the morphological image (Figure 3) compared to unirradiated cells (CTR group). In this group (200/5/C), further eight-fold irradiation had no effect, and ten-fold irradiation caused decreased cell adhesion to the substrate (Figure 2a). Increased adhesion of macrophages as a result of two applications with a continuous laser beam (day 3) was also observed in the case of cell irradiation with a laser at a power of 100 mW and a dose of 5 J/cm^2^ and 10 J/cm^2^ (100/5/C and 100/10/C groups) and a power of 200 mW and a dose of 10 J/cm^2^ (200/10/C group) (Figure 2a). In this situation, further cell irradiation had no effect or resulted in decreased cell adhesion, particularly on days 9 and 11 of macrophage culturing (100/5/C, 100/10/C, 200/5/C and 200/10/C groups) (Figure 2a).

With regard to pulsed beam treatment, for two- and six-fold irradiation of cells with a laser at a power of 200 mW and a dose of 5 J/cm^2^/well (200/5/P group), it was also found that cell adhesion to the substrate increased compared to the unirradiated cells (CTR group) (Figure 2b) and the density of macrophages was higher in the microscopic image (Figure 3). Further irradiation of macrophages using a laser with these parameters had no effect (day 9) or resulted in decreased cell adhesion to the substrate (day 11) (200/5/P group). An increase in cell adhesion was also found after two-fold irradiation with a pulsed laser beam of 100 mW and a dose of 5 J/cm^2^ (100/5/P group); however, further laser irradiation decreased cell adhesion compared to the unirradiated cells (CTR group). In turn, irradiation of cells with a laser with a power of 100 mW or 200 mW and a dose of 10 J/cm^2^ (100/10/P and 200/10/P groups) resulted in decreased cell adhesion compared to the unirradiated cells (CTR group) (Figure 2b). The results of adhesion were confirmed by the microscopic images of cells, in which it was shown that an increase in adhesion was accompanied by an increase in cell density, while a decrease in cell adhesion was accompanied by a decrease in cell density and cluster arrangement (Figure 3).

### 3.2. Level of AK Released by Macrophages Exposed to LLLT

**Figure 4:**
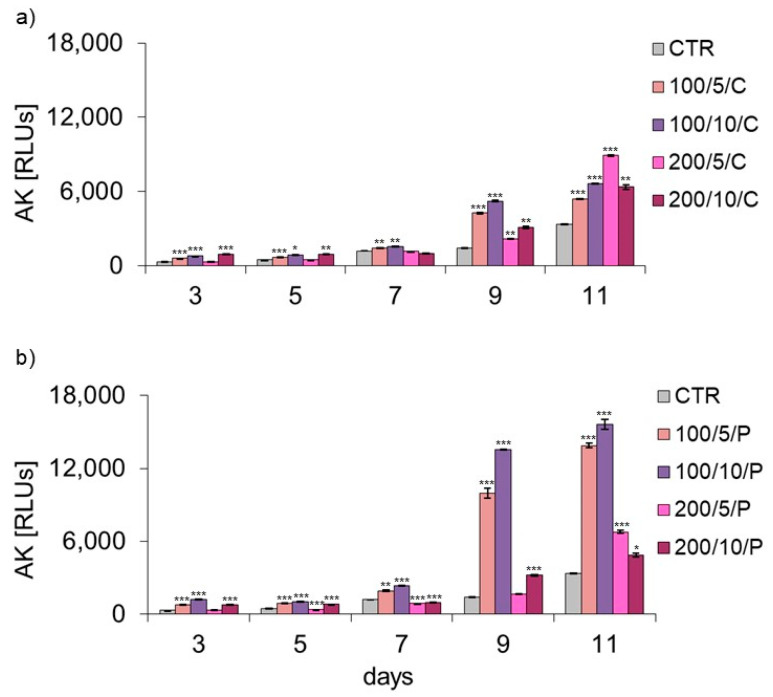
Effect of continuous (C) (**a**) and pulsed (P) (**b**) laser beam irradiation on cell adenylyl kinase (AK) release levels by macrophages of the RAW 264.7 cell line. Cells were cultured for a specified number of days and irradiated with a laser at powers of 100 or 200 mW and radiation doses of 5 or 10 J/cm^2^/well with cells. AK levels were measured on the next 3, 5, 7, 9 and 11 days of the experiment. RLUs—luminometer flux unit. Mean values ± SEM. *, **, ***—differences between cells irradiated with a laser of different parameters and cells not irradiated (CTR) (* *p* < 0.05, ** *p* < 0.01, *** *p* < 0.001).

Two-, four- and six-fold irradiation with a continuous laser beam of 200 mW and a dose of 5 J/cm^2^ (200/5/C group) did not affect the level of AK released by macrophages (Figure 4a). In turn, further eight- and ten-time applications of a laser beam with these parameters resulted in an increased AK level compared to the unirradiated cells (CTR group) (Figure 4a). Irradiation (regardless of the number of applications) with a continuous laser beam at a power of 100 mW and doses of 5 J/cm^2^ and 10 J/cm^2^ (100/5/C and 100/10/C groups) and 200 mW and a dose of 10 J/cm^2^ (200/10/C group) increased the AK level released by macrophages in these groups (Figure 4a) compared to the unirradiated cells (CTR group) (Figure 4a). Analysis of the effect of a pulsed laser beam at a power of 200 mW and dose of 5 J/cm^2^/well (200/5/P group) showed that four- and six-fold irradiation reduced the level of AK released by cells compared to the unirradiated macrophages (CTR group) (Figure 4b), although increasing the number of exposures to 10 also increased the level of released AK. Treatment with a pulsed laser beam at a power of 100 mW and doses of 5 and 10 J/cm^2^ (100/5/P and 100/10/P groups) and at a power of 200 mW and a dose of 10 J/cm^2^ (200/10/P group) induced an increase in the level of released AK (Figure 4b).

The results obtained from stage 1 of the study allowed us to select the irradiation parameters that had the most beneficial effect on the cells, causing an increase in cell adhesion/proliferation and no effect or decrease in the release of AK. For stage 2 of the study, the exposure parameters selected were continuous or pulsed laser beams at a power of 200 mW and a dose of 5 J/cm^2^ (200/5/C and 200/5/P groups).

### 3.3. Viability of Macrophages Irradiated with Continuous or Pulsed Laser Beams at a Power of 200 mW and Dose of 5 J/cm^2^

**Figure 5:**
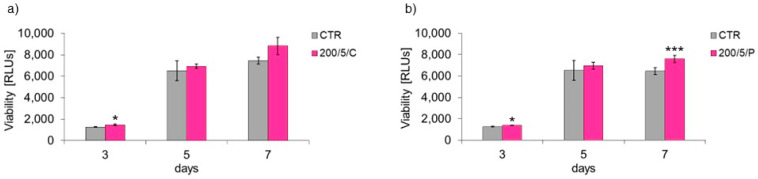
Effect of continuous (C) (**a**) and pulsed (P) (**b**) laser beam irradiation on viability macrophages of the RAW 264.7 cell line. Cells were cultured for a specified number of days and irradiated with a laser at a power of 200 mW and radiation dose of 5 J/cm^2^/well with cells. Viability was measured on the next 3, 5 and 7 days of the experiment. RLUs—luminometer flux unit. Mean values ± SEM. *, ***—differences between 200/5/C group and control group (CTR), and between 200/5/P and control group (CTR) (* *p* < 0.05, *** *p* < 0.001).

Two-fold irradiation with a continuous 200 mW laser beam and a dose of 5 J/cm^2^ (200/5/C group) caused an increase in macrophage viability compared to the unirradiated cells (CTR group) on day 3 of macrophage culturing (Figure 5a). Increasing the number of laser irradiations (four or six laser beam applications) had no effect on the viability of macrophages on days 5 and 7 of macrophage culturing. In turn, with regard to macrophages irradiated twice and six times with a pulsed laser beam of 200 mW and a dose of 5 J/cm^2^ (200/5/P group), increased cell viability was observed on days 3 and 7 of macrophage culturing compared to unirradiated cells (CTR group) (Figure 5b).

### 3.4. Effect of LLLT on the Secretory Activity of Macrophages Irradiated with a Pulsed Laser Beam at a Power of 200 mW and Dose of 5 J/cm^2^ (200/5/P Group)

**Figure 6:**
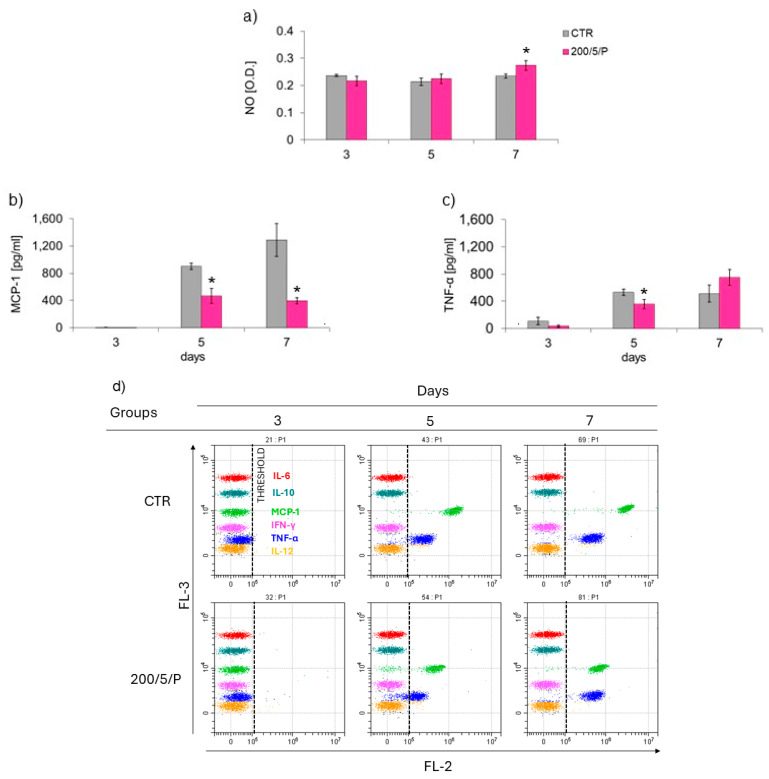
Effect of pulsed (P) laser beam irradiation on the levels of nitric oxide (NO) (**a**) and cytokines MCP-1 (**b**) and TNF-α (**c**) secreted by macrophages of the RAW 264.7 cell line, and example/representative dot plots of cytometric analysis of fluorescently labelled cytokines: IL-6, IL-10, MCP-1, IFN-γ, TNF-α and IL-12 (**d**). The y-axis (FL-3—bead channels) represents the different cytokines being measured, while the x-axis (FL-2—reporter channel) shows the fluorescence intensity, which is proportional to the concentration of each cytokine. The vertical dashed line indicates the threshold for a negative signal. Following separation of sera, cytokine content was evaluated by CBA, as described in the Materials and Methods. Cells were cultured for a specified number of days and irradiated with a laser at a power of 200 mW and radiation dose of 5 J/cm^2^/well with cells. Cytokine levels in supernatants were measured on the next 3, 5 and 7 days of the experiment. Mean values ± SM. (* for *p* < 0.05)—statistically significant differences between the group of irradiated (200/5/P) and non-irradiated cells (CTR group).

In the study, it was demonstrated that six-fold irradiation of macrophages resulted in increased NO secretion on day 7 of macrophage culturing, compared to the unirradiated cells (Figure 6a).

Four-fold laser irradiation of macrophages reduced the level of MCP-1 and TNF-α secretion (Figure 6b,c). Further six-fold laser irradiation decreased the secretion of TNF-α by macrophages compared to the unirradiated cells (CTR group) (Figure 6c). For the remaining cytokines measured, IFN-γ, IL-12p70, IL-6 and IL-10, their presence was not detected in any of the tested groups of cells (200/5/P and CTR groups), regardless of the tested time point.

**Figure 7:**
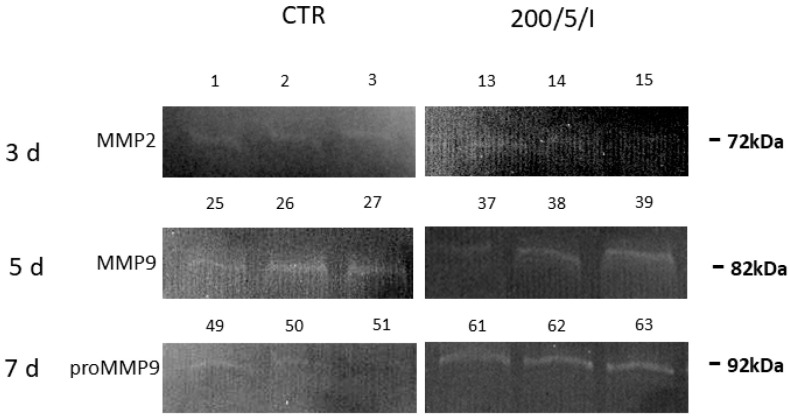
Representative gelatin zymogram of the RAW 264.7 cell line macrophage supernatant. The cells were cultured for a specified number of days (3, 5 and 7) and irradiated (twice, four and six times, respectively) with a laser at a power of 200 mW and radiation dose of 5 J/cm^2^/well with cells. The level of metalloproteinases was determined on the following days of the experiment: 3, 5 and 7.

In the trial, it was also shown that two- and four-fold laser irradiation of cells reduced the secretion of MMP-9 by macrophages compared to the unirradiated cells (CTR group) (Table 1). At all of the examined time points, there was no secretion or differences in the secretion of pro-MMP and MMP-2 by macrophages in the 200/5/P group, compared to the unirradiated cells (CTR group) (Table 1, Figure 7).

**Figure 8:**
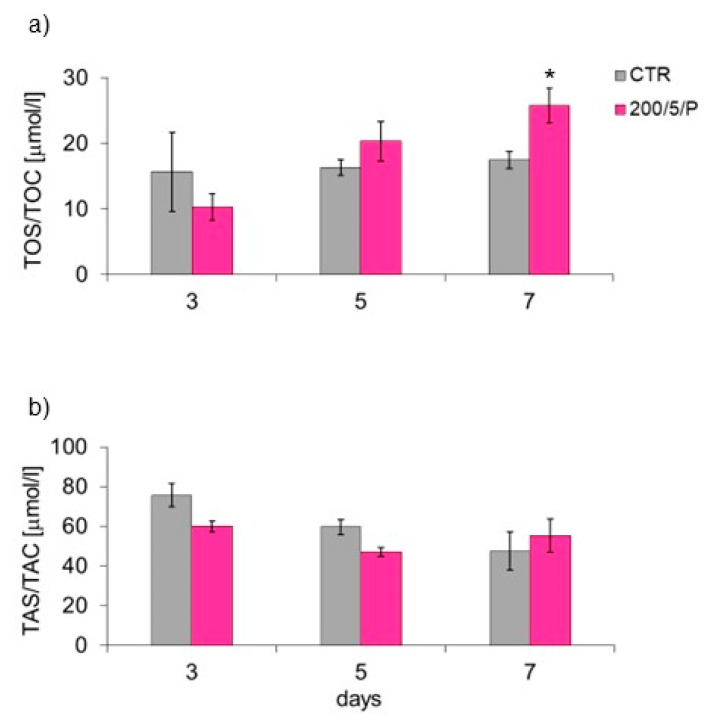
Effect of irradiation with a pulsed laser beam (P) on the oxidative (**a**) and antioxidant (**b**) potential of macrophages of the RAW 264.7 cell line. Cells were cultured for a specified number of days and irradiated with a laser with power of 200 mW and radiation dose of 5 J/cm^2^/well with cells. Oxidative potential (TOS/TOC) and antioxidant potential (TAS/TAC) were determined on the following days of the experiment: 3, 5 and 7. Mean values ± SEM. (* for *p* < 0.05)—statistically significant differences between the group of irradiated (200/5/P) and non-irradiated cells (CTR group).

Measurement of the TOS of macrophages showed that six-fold irradiation of the macrophages resulted in their increased total oxidative/capacitive status (TOS/TOC) compared to unirradiated cells (CTR group) (Figure 8a). However, for the TAS of irradiated macrophages (TAS/TAC), no differences were found compared to the unirradiated cells (CTR group) (Figure 8b).

## 4. Discussion

In many scientific studies, it has been shown that LLLT, as a result of contact with cells, may disturb homeostasis. It has been further demonstrated that LLLT can affect cell viability and secretory activity [2,3,33]. However, the authors of these works focused primarily on examining the effect of laser radiation on fibroblasts, keratinocytes or osteoblasts [6,8,34,35], while little is known about the effect of LLLT on the response of resting/tissue M0 macrophages, the activation of which is crucial in the course of healing [23].

In the present study, assays based on assessment of the adhesion and morphology of cells were used to identify the impact of laser irradiation. The results indicated significant differences in the ability of cells treated with LLLT to adhere/proliferate. The best effectiveness in promoting adhesion/proliferation was achieved when laser irradiation was applied at a power of 200 mW and a dose of 5 J/cm^2^ was used (200/5/C and 200/5/P groups). These results are confirmed by those of observations concerning the morphology of macrophages in this group, which suggested a clear increase in density and a tendency of proliferating cells to form clusters. Nonetheless, it should be noted that the beneficial effect of LLLT on cells was observed when they were irradiated up to six times. Further irradiation of the cells caused adhesion. The observed phenomenon probably resulted from the lack of a free surface for cell attachment and/or the lack of nutrients in the culture medium, and not the effect of increasing the number of irradiations. The phenomenon of decreased cell adhesion on the following days of the experiment was also found in the control group. Unfortunately, due to the lack of studies in the available literature regarding the effects of LLLT on macrophage adhesion/proliferation, it is difficult to compare the results obtained with those achieved in other studies. However, there are reports in which the effect is discussed of LLLT on the adhesion of other cell types. For example, Li et al. (2020) demonstrated that the use of LLLT at doses of 1.0 J/cm^2^, 2.0 J/cm^2^ and 4.0 J/cm^2^ increased the proliferation of human umbilical vein endothelial cells (HUVECs) [36]. Similarly, Sperandio et al. (2014) showed increased proliferation of HaCaT keratinocytes at an applied wavelength of 660 nm, power of 100 mW and laser energy densities of 3 J/cm^2^, 6 J/cm^2^ and 12 J/cm^2^ [37]. In turn, Basso et al. (2012) irradiated HGFs (human gingival fibroblasts) with a laser beam at a wavelength of 780 nm, power of 40 mW and doses of 0.5 J/cm^2^, 1.5 J/cm^2^, 3.5 J/cm^2^ and 7 J/cm^2^, noting that only two (0.5 J/cm^2^ and 3 J/cm^2^) of the tested six doses increased cell proliferation, while the remaining doses did not affect cell proliferation [35]. These authors did not observe any changes in the morphology of cells irradiated with LLLT either [35]. In the current study, the level of AK released from dead cells was also checked, while assessing the possible cytotoxic effect of LLLT on macrophages. Regardless of the method of radiation beam (continuous or pulsed) application, it was only in the group of cells irradiated with a laser at a power of 200 mW and dose of 5 J/cm^2^ that the level of AK released by the cells did not differ or was lower compared to the control group. Increasing the number of laser irradiations to eight and ten also increased the AK released from cells. The present study results indicate a safe/beneficial effect of resting macrophage irradiation with continuous or pulsed laser beams at a power of 200 mW and dose of 5 J/cm^2^ (200/5/C and 200/5/P groups). Therefore, these groups of cells were selected for further tests to assess cell viability. Additional testing in these groups exhibited increased viability of macrophages treated with a pulsed laser beam (200/5/P group), while continuous laser irradiation had no effect on improving macrophage viability (200/5/C group). Similar results were obtained by Silva et al. 2016, who irradiated inflammation-stimulated RAW 264.7 macrophages with two wavelengths, 660 nm and 880 nm, at a power of 100 mW and dose of 214 J/cm^2^, showing an increase in viability only when irradiated with a wavelength of 660 nm [38]. Also, according to Souza et al. (2014), inflammation-stimulated J774 macrophages irradiated with a laser at a wavelength of 660 nm, power of 15 mW and dose of 7.5 J/cm^2^ and wavelength of 780 nm, power of 70 mW and dose of 3 J/cm^2^ demonstrated increased viability compared to the group of macrophages not exposed to LLLT [39]. In turn, Song et al. (2021) studied the effect of a continuous laser beam at a wavelength of 810 nm and power of 80 mW on inflammation-stimulated RAW 264.7 macrophages, using laser radiation doses of 0.4 J/cm^2^, 1.2 J/cm^2^ and 2.4 J/cm^2^ [5]. In this case, only the dose of 2.4 J/cm^2^ significantly increased the viability of the tested cells. Similarly, Leden et al. (2013) examined the viability of stimulated BV2 macrophages irradiated with a laser at a wavelength of 808 nm, power of 50 mW and doses of 0.2 J/cm^2^, 4 J/cm^2^, 10 J/cm^2^ and 30 J/cm^2^, noting that only irradiation with doses of 4 J/cm^2^ and 30 J/cm^2^ resulted in viability [40].

In the presented research, it was shown that irradiation of resting macrophages with a pulsed laser beam (200/5/P group) caused increased NO secretion compared to non-irradiated cells (CTR group), but only on day 7 of culturing. Additionally, it was indicated that irradiation of cells with a pulsed laser beam increased the TOS of macrophages (also only on day 7 of culturing), but had no effect on the TAS of cells. According to Silva et al. (2016), monocytes and RAW 264.7 macrophages irradiated with a laser at a wavelength of 808 nm secreted more NO than cells irradiated at a wavelength of 660 nm [38]. An increase in NO secretion by stimulated BV2 macrophages was also demonstrated by Leden et al. (2013), who irradiated cells with a laser beam at 808 nm, power of 50 mW and doses of 4 J/cm^2^ and 30 J/cm^2^ [40]. These authors also observed that irradiation of cells with doses of0.2 J/cm^2^ and 10 J/cm^2^ at the same wavelength and laser power did not affect the secretion of NO by cells. NO is an important factor involved in the inflammatory response, produced and secreted mainly by classically polarised M1 macrophages [23]. Most authors agree that a decreased level of NO secreted by cells provides protection against excessive activation of M1 macrophages and is probably related to the defence of tissues at the inflammation site. In turn, high levels of NO and other pro-inflammatory mediators secreted by M1 macrophages may impair the proper phagocytosis of apoptotic cells and also directly damage host tissues [41]. Reacting with proteins, NO can cause nitrosylation of amino residues, impairing their function and causing a cytotoxic/apoptotic effect on surrounding cells. It can also stimulate the secretion of pro-inflammatory cytokines, exciting immune system cells and initiating inflammation [42]. Despite the increase in NO secretion by macrophages, the results of the current study suggest decreased secretion of cytokines TNF-α, MCP-1 and MMP-9 as a result of irradiation with a pulsed laser beam at 200 mW and a dose of 5 J/cm^2^. However, the tested samples did not contain other cytokines: IL-10, IL-12, IL-6 and IFN-γ.

A decrease in TNF-α secretion with a simultaneous increase in IL-10, IL-4 and IL-13 secretion was noted by Song et al. (2017), who irradiated inflammation-stimulated microglia cells with a laser at a wavelength of 810 nm, power of 150 mW and dose of 4.5 J/cm^2^ [2]. Similarly, Fernandes et al. (2015) observed a decrease in the secretion of TNF-α by J774 macrophages irradiated with a laser at a wavelength of 780 nm, power of 70 mW and doses of 2.6 J/cm^2^ and 660 nm, 15 mW, 7.5 J/cm^2^, while showing increased secretion of IL-6 by the cells irradiated with a 660 nm laser compared to unirradiated cells [43]. Souza et al. (2014) also examined the effect of laser irradiation at a wavelength of 780 nm, power of 70 mW and dose of 3 J/cm^2^ on the level of TNF-αsecreted by inflammation-stimulated macrophages. In this case, cell irradiation also reduced TNF-αsecretion [39]. In turn, Gavish et al. (2008) demonstrated that laser irradiation with a laser at a wavelength of 780 nm, power of 2 mW and dose of 2.2 J/cm^2^ decreased the secretion of MCP-1 by RAW 264.7 macrophages and had no effect on the secretion of IL-1β [44]. Different results were obtained by Leden et al. (2013). In their study, inflammation-stimulated BV2 macrophages, irradiated with a laser a wavelength of 808 nm, power of 50 mW and dose of 0.2 J/cm^2^ significantly increased the secretion of MCP-1, while higher doses of 4 J/cm^2^, 10 J/cm^2^ and 30 J/cm^2^ had no impact on the secretion of this cytokine by cells [40]. Li et al. (2020) measured the effect of laser irradiation at a wavelength of 810 nm, power of 150 mW and doses of 0.4 J/cm^2^, 4 J/cm^2^ and 10 J/cm^2^ on stimulated BMDM macrophages and observed that doses of 4 and 10 J/cm^2^, similarly to those used in the present study, inhibited the secretion of MCP-1, while a dose of 0.4 J/cm^2^ increased the secretion of MCP-1 and reduced IL-1β secretion [36]. Unlike in the current study, Li et al. (2020) also showed that the irradiation parameters used by the authors did not affect cytokine secretion by resting BMDM macrophages [36]. The effect of LLLT on the secretion of MMP-2 and MMP-9 described in the literature mainly concerns fibroblasts and osteoblasts [45,46]. For example, Ayuk et al. (2018) irradiated WS1 fibroblasts at two wavelengths, 660 nm (at a power of 108 mW) and 830 nm (at a power of 94 mW) and a dose of 5 J/cm^2^, showing that, regardless of the irradiation parameters used, the cells secreted less MMP-9 compared to the control [45]. In turn, Oliviera et al. (2017) irradiated MC3T3 osteoblasts with a laser at wavelengths of 660 nm and 780 nm, power of 20 mW and doses of 1.9 J/cm^2^ and 3.8 J/cm^2^, showing no effect of irradiation on the level of secreted MMP-9 and an increase in the secretion of MMP-2 by cells irradiated with a laser at a wavelength of 660 nm and dose of 1.9 J/cm^2^. The results of the presented study allow us to note that two- and four-fold laser irradiation of cells reduced the secretion of MMP-9 by macrophages compared to unirradiated cells [46].

## 5. Conclusions

The research results presented above indicate that the effect of LLLT on resting macrophages modulates their biological activity, and the intensity of biostimution depends on the irradiation parameters (wavelength, power, dose and method of laser beam application). The most beneficial radiation parameters for cells were irradiation with a pulsed laser beam at 200 mW of power and a dose of 5 J/cm^2^, which caused an increase in macrophage adhesion and viability, as well as an increase in NO secretion by macrophages and their TOS, with a simultaneous decrease in the secretions of TNF-α, MCP-1 and MMP-9 by cells. The results of the present study constitute a reference point and specific standard for planned, subsequent experiments aimed at assessing the effect of LLLT on the polarisation of macrophages in resting and inflamed states. The research results obtained within this project constitute a reference point and a kind of norm for the next stage of research. We plan to evaluate the synergistic effect of LLLT in combination with an active substance that can potentially support the healing process. The research will be conducted on resting macrophages and those polarised towards pro-inflammatory M1 macrophages.

## Figures and Tables

**Table 1:** Effect of irradiation with a pulsed (P) laser beam on the level of MMPs secreted by macrophages of the RAW 264.7 cell line. The average areas of the peaks corresponding to MMP2, pro-MMP9 and MMP9 in the samples were calculated and expressed as raw mean values (Raw Vol.).

Groups		CTR						200/5/P							
Number of laser applications		2		4		6		2		4		6			
Days		3		5		7		3		5		7		Comparison between groups 200/5/P v CTR	
		$\bar{x}$	SEM	$\bar{x}$	SEM	$\bar{x}$	SEM	$\bar{x}$	SEM	$\bar{x}$	SEM	$\bar{x}$	SEM	*p*	
Tested indicator	MMP-2	4900	1580	nd	-	nd	-	1158	466.87	nd	-	nd	-	day 3	-
														day 5	-
														day 7	-
	pro-MMP-9	nd	-	nd	-	2513	686	nd	-	419.52	286.3	16132	9230	day 3	-
														day 5	*p* = 0.144
														day 7	*p* = 0.143
	MMP-9	nd	-	4498	280	nd	-	147.06	61.34	2116.8	574	nd	-	day 3	*p* = 0.050 *
														day 5	*p* = 0.024 *
														day 7	-

The cells were cultured for a specified number of days (3, 5 and 7) and irradiated (twice, four and six times, respectively) at a laser with power of 200 mW and radiation dose of 5 J/cm^2^/well with cells. The level of metalloproteinases was determined on the following days of the experiment: 3, 5 and 7. Mean values ± SEM; nd—no metalloproteinases were detected. (* for *p* < 0.05)—statistically significant differences between the group of irradiated (200/5/P) and non-irradiated cells (CTR group) on individual days of the experiment (3, 5 and 7).

## Data Availability

The raw data supporting the conclusions of this article will be made available by the authors upon reasonable request.
